# Supplementary material for: An Intelligent Fire-Protection Coating Based on Ammonium Polyphosphate/Epoxy Composites and Laser-Induced Graphene
Source: Polymers (Basel). 2021 Mar 23;13(6):984. doi: 10.3390/polym13060984 (PMC8004711; doi:10.3390/polym13060984)
Supplement: Supplementary file 1 [file polymers-13-00984-s001.pdf]

Supporting Information

An Intelligent Fire Protection Coating Based on Ammonium Polyphosphate/Epoxy Composites and Laser Induced Graphene

Weiwei Yang, Ying Liu, Jie Wei, Xueli Li, Nianhua Li and Jiping Liu *

School of Materials Science and Engineering, Beijing Institute of Technology, Beijing 100081, China; yangweiwei0811@163.com (W.Y.); yingliu@bit.edu.cn (Y.L.); jie_weiwj@163.com (J.W.); 15733185216@163.com (X.L.); 17888818050@163.com (N.L.)

* Correspondence: liujp@bit.edu.cn; Tel.: +13910788891

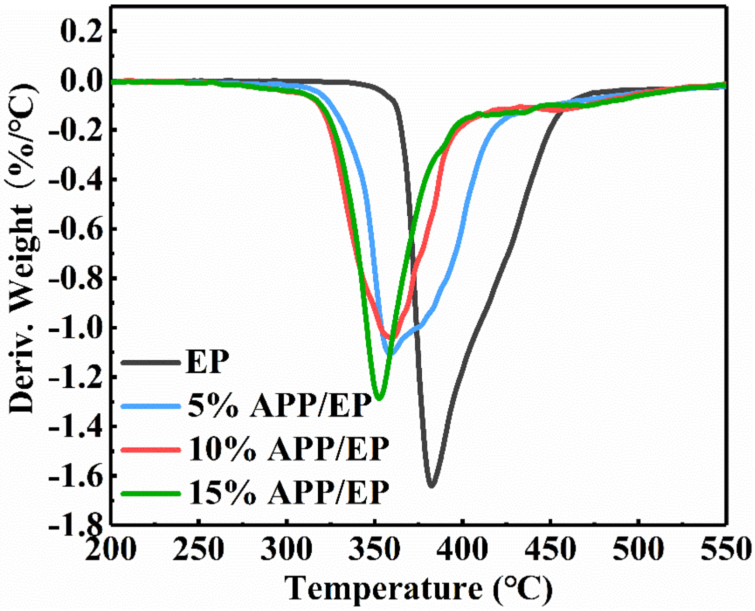

Figure S1. DTG curves of EP and APP/EP.

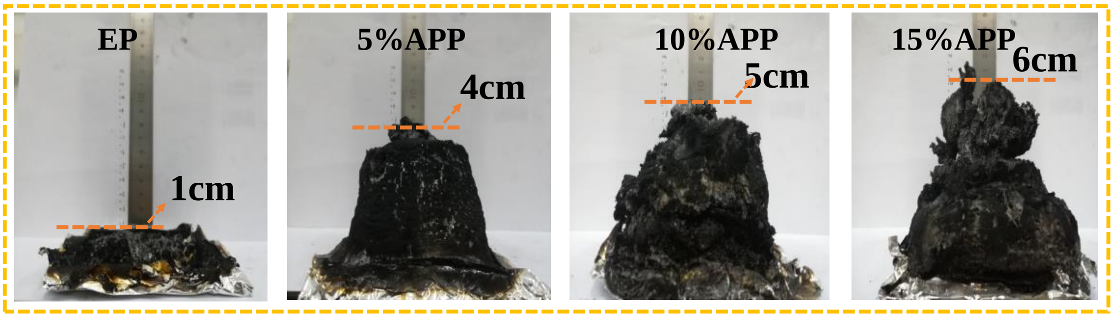

Figure S2. The residue carbon of different APP content in EP.

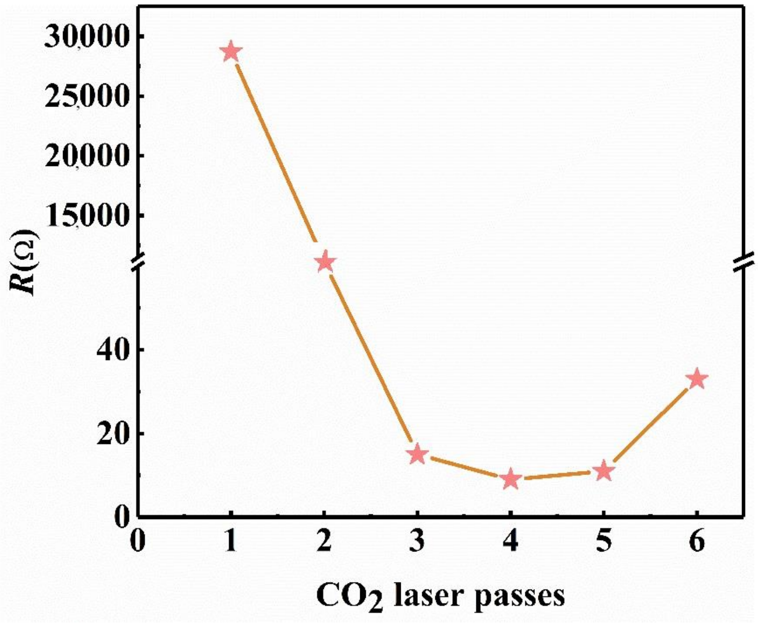

Figure S3. Resistance of LIG samples (1×1 cm²) prepared by different laser passes.

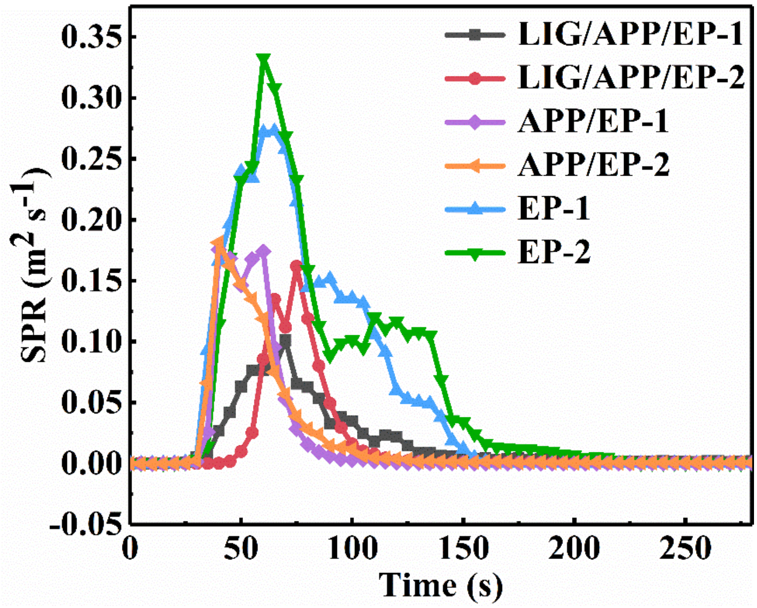

Figure S4. SPR curves of EP, APP/EP and LIG/APP/EP.

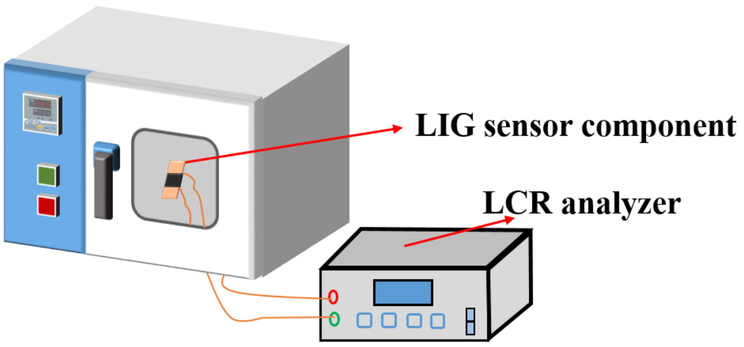

Figure S5. The schematic diagram of LIG sensor test device.

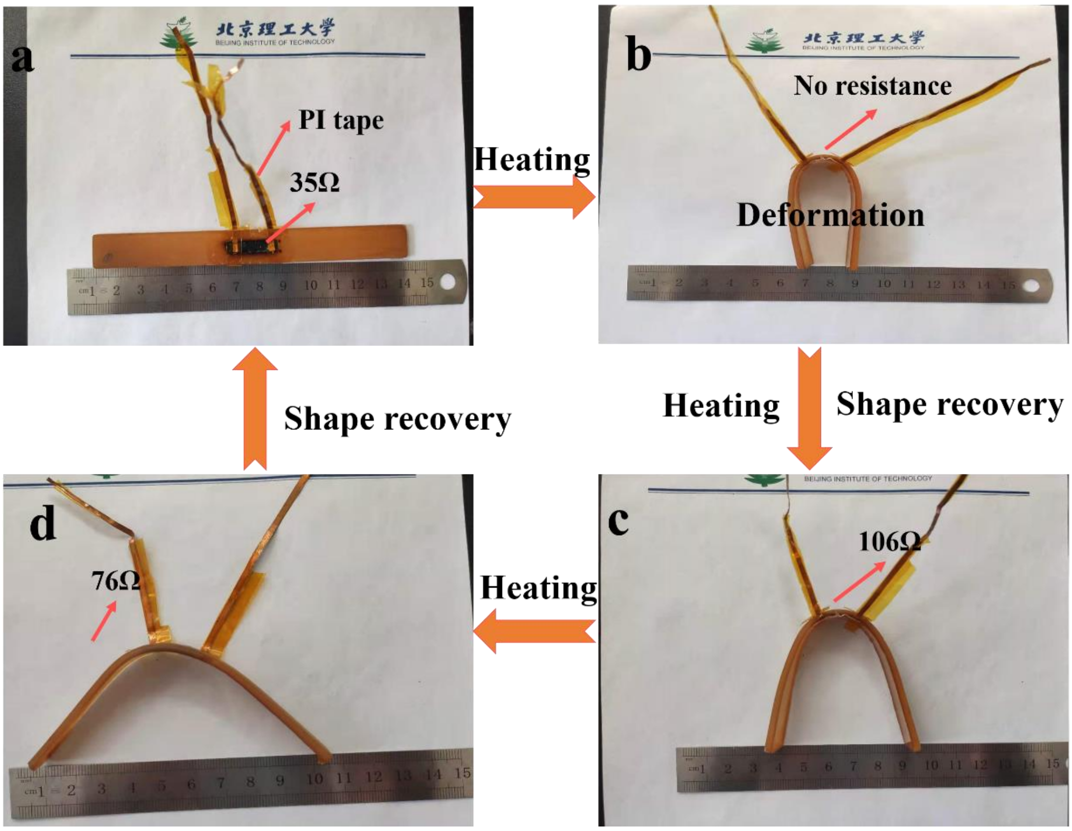

Figure S6. The resistance of LIG sensor according shape memory effect.
